# Oolong Tea Consumption and the Risk of Oral Squamous Cell Carcinoma: A Propensity Score-Based Analysis in Southeast China

**DOI:** 10.3389/fnut.2022.928840

**Published:** 2022-07-07

**Authors:** Qingrong Deng, Yuying Wu, Xiaoying Hu, Huiqing Wu, Mengzhu Guo, Yimin Lin, Menglin Yu, Wenwen Huang, Yuxuan Wu, Lisong Lin, Yu Qiu, Jing Wang, Baochang He, Fa Chen

**Affiliations:** ^1^Department of Epidemiology and Health Statistics, School of Public Health, Fujian Medical University, Fuzhou, China; ^2^Key Laboratory of Ministry of Education for Gastrointestinal Cancer, Fujian Medical University, Fuzhou, China; ^3^Department of Oral and Maxillofacial Surgery, The First Affiliated Hospital of Fujian Medical University, Fuzhou, China; ^4^Laboratory Center, The Major Subject of Environment and Health of Fujian Key Universities, School of Public Health, Fujian Medical University, Fuzhou, China

**Keywords:** oolong tea consumption, OSCC, propensity score analyses, oral hygiene, risk assessment

## Abstract

Oolong tea is one of the world's most popular non-alcoholic beverages, particularly in coastal Southeast China. Hitherto, epidemiological studies on the association between oolong tea consumption and the risk of patients with oral squamous cell carcinoma (OSCC) are very limited. This study aimed to evaluate the potential effect of oolong tea consumption on OSCC risk in Southeast China. From January 2010 to October 2020, face-to-face interviews were conducted for 744 newly diagnosed OSCC patients and 1,029 healthy controls to collect information on demographics, oolong tea consumption behaviors, and other lifestyle factors. Propensity score matching (PSM), inverse probability of treatment weight (IPTW), and stabilized inverse probability of treatment weight (SIPTW) were utilized to minimize confounding effects. Multivariate, conditional, and weighted logistic regression was used to evaluate the associations of oolong tea consumption behaviors with OSCC risk. Participants who drank oolong tea showed a lower risk of OSCC when compared to their non-drink counterparts [PSM population, OR (95%CI): 0.69 (0.49–0.97); SIPTW population, OR (95%CI): 0.74 (0.58–0.94)]. Moreover, the reduced risk was found to be significantly associated with certain tea-drinking habits (consumed amount over 500 mL per day, a duration of <20 years, age at initiation older than 30 years, and warm and moderately concentrated tea). Similar results were yielded in the sensitivity analyses (Multivariate adjustment and the IPTW analysis). Furthermore, subgroup analysis revealed that the negative association of oolong tea drinking with OSCC risk was more evident among those with poor oral hygiene. This study provides supportive evidence that oolong tea consumption may have a potentially beneficial effect in preventing OSCC, especially for those with poor oral hygiene.

## Introduction

Oral cancer is the most typical tumor in the head and neck, with oral squamous cell carcinoma (OSCC) accounting for nearly 90% of its pathological classifications (1). The incidence of oral cancer has shown a gradual upward trend in recent years, which makes it a growing concern for the global public, particularly in developing nations (2, 3). It was estimated that the worldwide number of new oral cancer will reach 421,907 by 2025 (4).

Tea has been recognized as one of the world's most renowned beverages, with common varieties including green tea, black tea, and oolong tea (5). Among which, oolong tea is a traditional type of tea origin in southeast China, very popular throughout Asia (6). Accumulating evidence has indicated that oolong tea is rich in polyphenols, flavonoids, and other chemical compounds, which has been posing an increasing interest worldwide (7). Previous studies revealed that oolong tea consumption was associated with a lower risk of numerous chronic diseases, such as hypertension (8), dyslipidemia (9), ischemic stroke (10), and cardiovascular diseases (11). There are also some reports on the effect of oolong tea consumption on several cancers including esophageal cancer (12), ovarian cancer (13), and nasopharyngeal cancer (14). However, to date, research on the potential association between oolong tea consumption and OSCC risk is very limited. Although our previous study suggests a negative association between drinking tea and oral cancer risk (15, 16), continued research into the role of oolong tea consumption is worthy of further exploration. Therefore, the purpose of this study was to investigate the relationship between oolong tea drinking behaviors and oral cancer risk using propensity score analyses (including propensity score matching, PSM; inverse probability of treatment weight, IPTW; and stabilized inverse probability of treatment weight, SIPTW) to minimize the potential confounding effects.

## Materials and Methods

### Study Population

The study was conducted on a hospital-based case-control design which was conducted from January 2010 to October 2020 in Fujian Province, China. A total of 744 patients with newly diagnosed cases of OSCC were recruited from the First Affiliated Hospital of Fujian Medical University, whereas 1,029 healthy controls were registered from the hospital's physical examination center during the same period. Potential research participants who satisfied all inclusion and exclusion criteria and provided written informed consent to participate were enrolled. Furthermore, the inclusion and exclusion criteria were previously described in detail (15). Briefly, all the participants were (1) aged 18–90 years old; and (2) capable of answering questions effectively. Cases were defined as histologically confirmed primary OSCC with no history of chemotherapy or radiation. Controls were classified as healthy people with no history of ophthalmic, cutaneous, respiratory, gastrointestinal, or oncological disorders. This study was approved by the Institutional Review Board of Fujian Medical University (Fuzhou, China) and carried out in conformity with the ethical criteria outlined in the Helsinki Declaration in 1964.

### Data Collection

After obtaining written informed consent from research respondents, data were collected by trained professional investigators using an interview-based structured questionnaire. The information obtained included (1) demographics: age, gender, occupation, education level, residence, body height, and weight, etc.; (2) self-reported lifestyle habits: alcohol consumption (yes or no), tobacco smoking (yes or no), and oolong tea drinking-related habits; (3) common dietary intake frequency (Red meat, vegetable intake and fruits intake); and (4) oral hygiene indicators: tooth brushing/day, the numbers of missing teeth, duration of wearing dentures (years), regular dental visits (no/yes), and whether they had oral ulcers (no/ yes). The age was divided into non-elderly (<60 years) and elderly groups (≥60 years) according to the definition of elderly of the United Nations (1). And the dietary intake frequency (red meat, vegetable intake, and fruit intake) was grouped into two groups according to the median of the control group in the overall population. Red meat intake: <3 and ≥3 times/week. Vegetable intake: <2 and ≥2 times/day. Fruit intake: <3 and ≥3 times/week. We constructed a comprehensive index for assessing oral health conditions that are based on the five indicators above. The details have been published in a previous article (17) and were summarized in Supplementary Table 1.

Those who smoked more than 100 cigarettes throughout their lifetime were defined as smokers (18). And those that drank alcohol had at least one drink every week for at least 6 months (15). Oolong tea drinkers were defined as those who consume at least one cup of tea every week for at least 6 months. In this study, there were only 119 oolong tea drinkers who did not smoke or drink alcohol (90 in the control group and 29 in the OSCC group). The detailed data and interaction between tea consumption and smoking or alcohol drinking were presented in Supplementary Table 2.

The following are details on oolong tea drinking habits: (1) Oolong tea drinking history (yes or no); (2) Years of tea-drinking (years); (3) Average daily tea consumption (ml/day); (4) age reported started tea-drinking (in years); (5) Tea temperature (non-drinker/warm/hot); and (6) Tea concentration (light/moderate/strong). It relied on the subjects' assessment of the temperature of the tea. To minimize miss classification, we set a judgment criterion based on the average time from mixing the tea leaves with boiling water to the time of tea drinking. The temperature of tea drinking was classified as very hot (<1 min), hot (1–5 min), warm (5–10 min), and cold tea (more than 10 min) (19). The number of individuals who drink cool tea or very hot was too small to include in this analysis; and the concentration of tea was evaluated based on the volume filled by the brewed tea leaves in the cup (light, <25% of the cup; moderate, 25–50% of the cup; and strong, >50% of the cup) (15). Additionally, other oolong tea drinking habits (such as amount, duration, and age at initiation) were set as categorical variables according to the median of the control group who had a history of drinking oolong tea in overall population. The details are listed as followed: average daily intake amount was grouped into three groups (never drinking, <500 ml/d, ≥500 ml/d); duration of tea consumption was grouped into three groups (never drinking, <20 years, ≥20 years); age at onset of regular drinking was also classified into three groups (never drinking, <30 years, ≥30 years).

### Statistical Analysis

In the overall data, Chi-square or Fisher's exact tests were used to compare baseline features between OSCC patients and healthy controls. Propensity score analyses, including propensity score matching (PSM), inverse probability of treatment weight (IPTW), and stabilized inverse probability of treatment weight (SIPTW), were used to minimize selection bias and balance baseline differences and other confounding factors. The matching ratio in PSM was 1:1, and the caliper was 0.02, with 487 patients correctly matched with 487 healthy controls. Based on the PSM, IPTW is then calculated with the estimated propensity score. SPTW was also used to minimize sample size inflation and ensure accurate variance estimates (20, 21). Group differences were measured using standardized mean differences (SMD), with an SMD value of 0.1 considered balanced after the matching. To determine the associations between oolong tea-drinking habits and OSCC risk, we utilized multivariate logistic regression (in unmatched data), conditional logistic regression (in PSM data), and weighted logistic regression (in IPTW and SIPTW data). A two-tailed *P*-value of 0.05 was deemed statistically significant. R software version 4.0.3 was used for all analyses. For propensity scoring and matching analysis, the “MatchIt” package was used, and the “forestploter” package was used to visualize the stratified analysis results.

## Results

**Figure 1:**
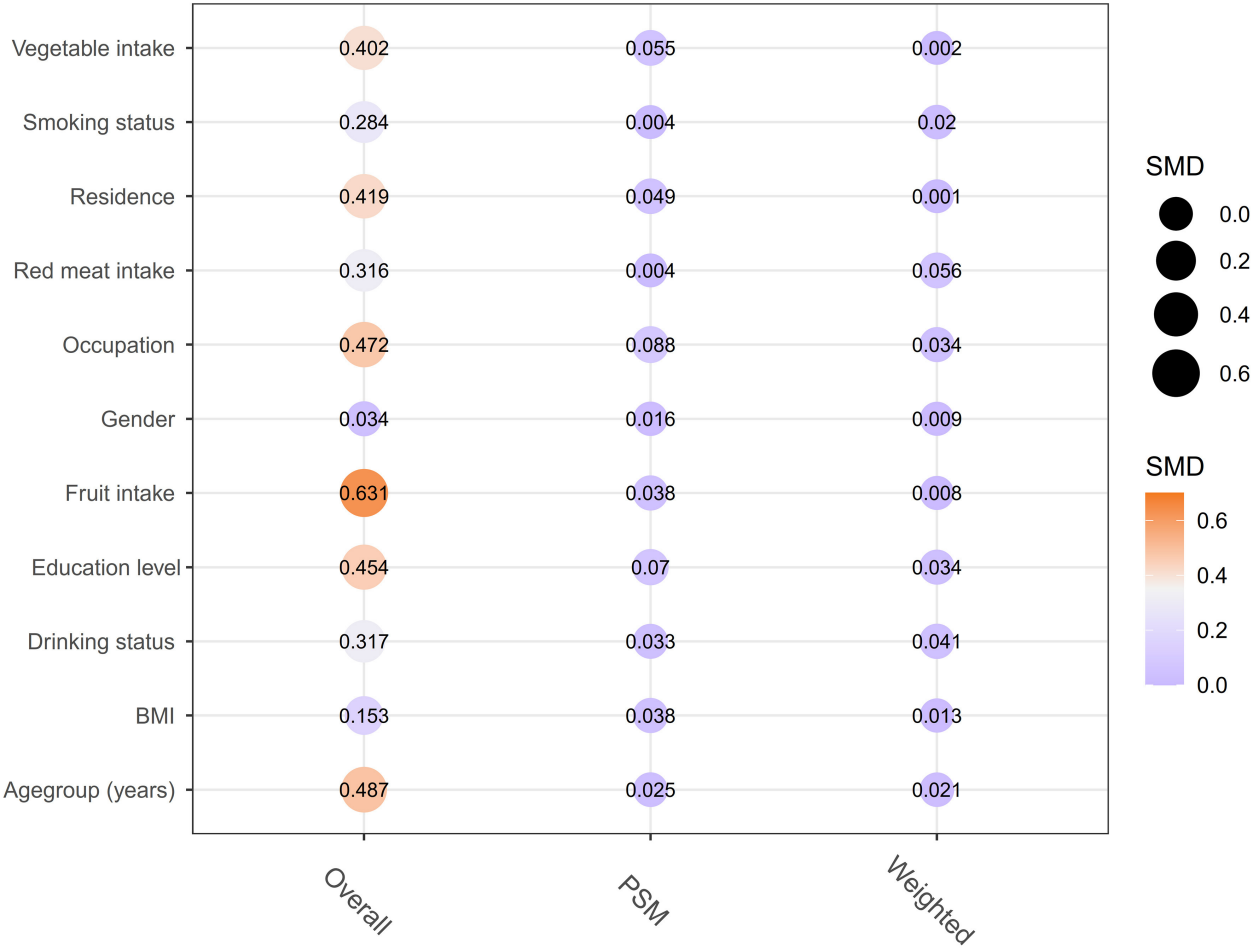
Comparison of the distribution of matching factors between cases and controls before and after matching, Group differences were assessed using standardized mean differences (SMD), with an SMD value of 0.1 considered balanced.

This observational case-control research comprised 744 OSCC patients and 1,029 healthy controls. A baseline summary of pre-and post-matching participant demographics (age, gender, occupation, education level, BMI, and residence), lifestyle habits (smoking and alcohol consumption), and consumption frequency of red meat, vegetables, and fruits are presented in Table 1 (population after PSM and SIPTW) and Supplementary Table 3 (overall population and population after IPTW). Before matching analysis, imbalances were noted between the case and control subjects in the collected 11 variables except for gender (*P* < 0.05). After propensity matching adjustment (PSM, IPTW, and SIPTW analysis), all the distributions of observed covariates were comparable (SMD < 0.1, Table 1 and Supplementary Table 3 and Figure 1).

**Table 1:** Baseline characteristics of case and control groups after propensity score analyses.

**Variables**		**PSM population**			**SIPTW population**		
		**Control (%)**	**Case (%)**	**SMD**	**Control (%)**	**Case (%)**	**SMD**
*N*		487	487		1,038.0	748.0	
Gender	Male	265 (54.4)	261 (53.6)	0.016	572.7 (55.2)	416.1 (55.6)	0.009
	Female	222 (45.6)	226 (46.4)		465.3 (44.8)	331.9 (44.4)	
Age (years)	<60	274 (56.3)	280 (57.5)	0.025	622.6 (60.0)	456.3 (61.0)	0.021
	≥60	213 (43.7)	207 (42.5)		415.4 (40.0)	291.7 (39.0)	
Occupation	Farmer	125 (25.7)	140 (28.7)	0.088	266.0 (25.6)	184.7 (24.7)	0.034
	Worker	78 (16.0)	84 (17.2)		166.7 (16.1)	114.7 (15.3)	
	Office worker and others	284 (58.3)	263 (54.1)		605.3 (58.3)	448.6 (60.0)	
Education level	Illiteracy	55 (11.3)	66 (13.6)	0.070	118.7 (11.4)	89.4 (11.9)	0.034
	Primary-middle school	282 (57.9)	278 (57.0)		577.3 (55.6)	403.6 (54.0)	
	High school and above	150 (30.8)	143 (29.4)		342.0 (33.0)	255.0 (34.1)	
BMI	18.5–23.9	291 (59.8)	300 (61.6)	0.038	620.8 (59.8)	451.9 (60.4)	0.013
	<18.5 or ≥24	196 (40.2)	187 (38.4)		417.2 (40.2)	296.1 (39.6)	
Residence	Rural	221 (45.4)	233 (47.8)	0.049	464.9 (44.8)	334.8 (44.8)	<0.001
	Urban	266 (54.6)	254 (52.2)		573.1 (55.2)	413.2 (55.2)	
Smoking status	No	325 (66.7)	324 (66.5)	0.004	701.2 (67.6)	512.3 (68.5)	0.020
	Yes	162 (33.3)	163 (33.5)		336.8 (32.4)	235.7 (31.5)	
Drinking status	No	369 (75.8)	362 (74.3)	0.033	784.6 (75.6)	578.2 (77.3)	0.041
	Yes	118 (24.2)	125 (25.7)		253.5 (24.4)	169.8 (22.7)	
Red meat intake	<3 times	280 (57.5)	281 (57.7)	0.004	577.0 (55.6)	394.8 (52.8)	0.056
(per week)	≥3 times	207 (42.5)	206 (42.3)		461.0 (44.4)	353.2 (47.2)	
Vegetable intake	<2 times	185 (38.0)	172 (35.3)	0.055	358.0 (34.5)	257.4 (34.4)	0.002
(per day)	≥2 times	302 (62.0)	315 (64.7)		680.0 (65.5)	490.6 (65.6)	
Fruit intake	<3 times	290 (59.5)	281 (57.7)	0.038	536.2 (51.7)	383.3 (51.2)	0.008
(per week)	≥3 times	197 (40.5)	206 (42.3)		501.8 (48.3)	364.7 (48.8)	

*PSM, propensity score matching; SIPTW, stabilized inverse probability of treatment weight; SMD, standardized mean differences*.

Table 2 presents the relationships between oolong tea-drinking habits and the risk of OSCC in PSM and SIPTW populations. Reduced risk of OSCC was observed among participants who had the consumption of oolong tea as compared to non-drinkers [PSM population, OR (95%CI): 0.69 (0.49–0.97); SIPTW population, OR (95%CI): 0.74 (0.58–0.94)]. Moreover, oolong tea drinkers who consumed over 500 mL per day had a statistically significant decrease in OSCC risk with the OR (95%CI) being 0.59 (0.38–0.92) in PSM analysis and 0.71 (0.57–0.96) in SIPTW analysis. Additionally, those who had a duration of oolong tea consumption of <20 years and those who started drinking tea at an age older than 30 years were less likely to develop OSCC than non-tea drinkers. Of note, both temperature and concentration of oolong tea were correlated with the risk of OSCC. In PSM data, there is a lower risk of OSCC for oolong tea drinkers who prefer warm and moderately concentrated tea over non-tea drinkers [Tea temperature: warm vs. never drinking, OR (95% CI): 0.56 (0.33–0.93); Tea concentration: moderate vs. never drinking, OR (95% CI): 0.56 (0.33–0.93)]. Similar relationship patterns were also identified in SIPTW data [Tea temperature: warm vs. never drinking, OR (95% CI): 0.70 (0.50–0.99); Tea concentration: moderate vs. never drinking, OR (95% CI): 0.64 (0.46–0.90)]. Subsequently, we performed sensitivity analyses on the overall population and the IPTW population to assess the stability of the results, and comparable results were obtained (Supplementary Table 4).

**Table 2:** The relationship between oolong tea-drinking habits and OSCC risk after propensity score analyses.

**Variables**	**PSM population**			***P* for trend**	**SIPTW population**			***P* for trend**
	**Control**	**Case**	**OR (95%CI)**		**Control**	**Case**	**OR (95%CI)**	
**Oolong tea consumption**								
No	386 (79.26)	411 (84.40)	1.00		817.5 (78.75)	623.5 (83.36)	1.00	
Yes	101 (20.74)	76 (15.60)	**0.69 (0.49–0.97)**		220.6 (21.25)	124.4 (16.64)	**0.74 (0.58–0.94)**	
**Average daily intake (ml/d)**				0.025				0.001
Never drinking	386 (79.26)	411 (84.40)	1.00		817.5 (78.75)	623.5 (83.36)	1.00	
<500	36 (7.39)	32 (6.57)	0.84 (0.51–1.36)		86.3 (8.31)	51.7 (6.91)	0.79 (0.55–1.13)	
≥500	65 (13.35)	44 (9.03)	**0.59 (0.38–0.92)**		134.3 (12.94)	72.8 (9.73)	**0.71 (0.52–0.96)**	
**Duration of tea consumption (years)**				0.275				0.180
Never drinking	386 (79.26)	411 (84.40)	1.00		817.5 (78.75)	623.5 (83.36)	1.00	
<20	51 (10.47)	23 (4.72)	**0.41 (0.24–0.70)**		103.7 (9.99)	42.4 (5.67)	**0.54 (0.37–0.78)**	
≥20	50 (10.27)	53 (10.88)	0.96 (0.63–1.47)		116.9 (11.26)	82.0 (10.97)	0.92 (0.68–1.24)	
**Age at onset of regular drinking (years)**				0.002				<0.001
Never drinking	386 (79.26)	411 (84.40)	1.00		817.5 (78.75)	623.5 (83.36)	1.00	
<30	38 (7.80)	48 (9.85)	1.12 (0.71–1.77)		87.6 (8.44)	78.7 (10.52)	1.18 (0.85–1.63)	
≥30	63 (12.94)	28 (5.75)	**0.42 (0.26–0.68)**		133.0 (12.81)	45.7 (6.12)	**0.45 (0.32–0.64)**	
**Tea temperature**				0.103				0.010
Never drinking	386 (79.26)	411 (84.40)	1.00		817.5 (78.75)	623.5 (83.36)	1.00	
Warm	45 (9.24)	28 (5.75)	**0.56 (0.33–0.93)**		102.3 (9.85)	54.8 (7.32)	**0.70 (0.50–0.99)**	
Hot	56 (11.50)	48 (9.85)	0.78 (0.52–1.18)		118.3 (11.40)	69.7 (9.32)	0.77 (0.56–1.06)	
**Tea concentration**				0.156				0.020
Never drinking	386 (79.26)	411 (84.40)	1.00		817.5 (78.75)	623.5 (83.36)	1.00	
Light	29 (5.95)	20 (4.10)	0.64 (0.36–1.16)		50.8 (4.89)	31.7 (4.24)	0.82 (0.52–1.29)	
Moderate	54 (11.09)	33 (6.78)	**0.56 (0.35–0.90)**		119.5 (11.52)	58.7 (7.85)	**0.64 (0.46–0.90)**	
Strong	18 (3.70)	23 (4.72)	1.20 (0.61–2.37)		50.2 (4.84)	34.0 (4.55)	0.89 (0.57–1.39)	

*OSCC, oral squamous cell carcinoma; PSM, propensity score matching; SIPTW, stabilized inverse probability of treatment weight; OR (95% CI), the odds ratio and its 95% confidence interval. The significant ORs (95% CI) was bolded for ease of viewing*.

**Figure 2:**
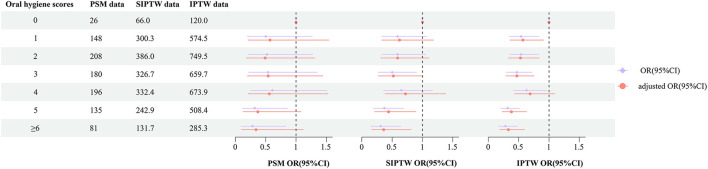
Associations of oolong tea consumption and the risk of OSCC, stratified by different oral hygiene scores. Adjusted OR (95% CI) was calculated by adjusting for age, gender, occupation, education level, BMI, residence, smoking, and alcohol consumption, consumption frequency of red meat, vegetables, and fruits.

Based on the oral hygiene scores, the subjects were divided into subgroups for further analysis. A greater reduction in OSCC risk with oolong tea drinking was observed among those with poor oral hygiene compared to non-drinkers with good oral hygiene. Of note, as the sample size increases (PSM population to IPTW population), the observed inverse association with OSCC seems to be more pronounced (Figure 2).

## Discussion

This hospital-based case-control study sought to shed light on the relationship between oolong tea consumption and OSCC risk using three powerful propensity score analyses. Overall, our results support a beneficial effect of oolong tea consumption on the reduced risk of OSCC. Furthermore, OSCC risk was found to be significantly associated with certain tea-drinking habits (amount, duration, age at initiation, temperature, and concentration). Of note, the possible preventive benefits of oolong tea consumption against OSCC were more prominent in individuals with poor oral hygiene.

In the present study, we found the consumption of oolong tea reduced the risk of OSCC by 25–30%, which corresponded to the results of our previous studies (15, 16). In addition, the risk can be further reduced by a higher dose of tea exposure (more than 500 ml/day). Zhou et al. (22). recently published a meta-analysis and systematic review also showing that increasing one cup of tea per day reduces the incidence of oral cancer by 6.2%. More than 100 chemical components have been isolated and identified from oolong tea, among which polyphenols are the most significant ones (23, 24). The polyphenols contain multiple functional components, such as (-)-epigallocatechin gallate (EGCG) and theaflavin (TF) (25, 26), which were reported to have anti-inflammatory and antioxidant effects, and other biological properties (5, 27). Also, multiple *in vitro and in vivo* studies have indicated that several oolong tea polyphenol extracts could induce apoptosis or proliferation of cancer cells, including oral cancer cells, leading to tumor growth inhibition (28–31).

In the present study, individuals who had consumed oolong tea for <20 years and those who began drinking tea at an age ≥30 years were less likely to develop OSCC, indicating the long-term benefits of oolong tea, when compared to non-oolong-tea drinkers. It is hypothesized that the possible explanation is that the incidence of OSCC grows with increasing age, and young individuals accounted for more in the group with <20 years of tea drinking, while the elderly accounted for more in the group with ≥20 years of tea drinking. Certainly, this hypothesis will require further investigation in future studies.

In the present study, we also found a significantly lower risk of oral cancer among warm tea drinkers compared to non-tea drinkers, while this protective effect tended to diminish among hot tea drinkers (not reaching statistical significance). Recently, the International Agency for Research on Cancer (IARC) classified drinking very hot beverages above 65°C as “probably carcinogenic to humans” (32). According to the most recent study by Ernst et al. (33), hot beverages would increase the cell division rate in the oral mucosa at temperatures above 60°C, leading to cytotoxic effects and increased risk of cancer. Drinking moderately concentrated tea would be most beneficial to reducing OSCC risk in our study (Table 2 and Supplementary Table 4). However, the literature is controversial concerning the optimal tea-drinking concentration. Dose-response analyses of one meta-analysis suggested that with the concentration of tea consumption increased, the risk of oral cancer decreased (22). However, there have been other studies showing that fluoride found in oolong tea helps to prevent dental caries and promote healthy bone growth, but excess fluoride could lead to detrimental health problems in humans, especially fluorosis of the teeth and skeletal fluorosis (7).

It is crucial to highlight that the preventative advantages of oolong tea drinking may vary on oral hygiene status, with individuals with poor oral hygiene experiencing stronger protective benefits. According to Yoo et al. (34), oolong tea extract has an antibacterial effect on oral streptococci such as *Streptococcus mutans* and *Streptococcus sobrinus*. In polyphenols, notably catechin, epigallocatechin-3-gallate selectively inhibits the development and adhesion of periodontopathogens (35). These findings imply that long-term consumption of oolong tea may be effective in mitigating the negative effects of poor oral hygiene on OSCC, which has significant public health implications.

There are certain merits to this study. Our findings represent the first comprehensive analysis of oolong tea consumption and related habits (including amount, duration, age at initiation, temperature, and concentration) and OSCC risk by utilizing traditional multivariate logistic regression (Supplementary Table 4) and advanced propensity score analyses (PSM, IPTW, and SIPTW analyses, Table 2 and Supplementary Table 4). However, several limitations of our investigation were unavoidable. Firstly, despite the adjustment of potential influencing factors to minimize this effect when performing the stratified analysis of oral hygiene scores, the balance between groups may still be disrupted, and future studies using other statistical methods (such as propensity score stratification) are needed. Secondly, it's difficult to accurately correlate each subject with the true situation since some subjects consumed more than one type of tea, while only the main tea consumed was recorded in the survey, and differences in composition between oolong teas of different origins may further obscure the true association between tea drinking and the risk of OSCC. Third, considering the relatively small sample size of oolong tea drinkers who neither smoked nor consumed alcohol in this study, we are unable to further analyze the associations of oolong tea consumption habits (amount, duration, age at initiation, and concentration) with OSCC risk among this population. the results must be validated in larger cohorts. Further research with larger sample size is warranted to confirm these results.

## Conclusion

In conclusion, this study suggests an inverse association between oolong tea consumption and the risk of OSCC, especially for those with poor oral hygiene conditions. These findings may provide an additional understanding of the beneficial role of oolong tea consumption in decreasing the risk of OSCC, which has public health implications for oral cancer prevention.

## Data Availability Statement

The original contributions presented in the study are included in the article/Supplementary Material, further inquiries can be directed to the corresponding author.

## Ethics Statement

This study was approved by the Institutional Review Board of Fujian Medical University (Fuzhou, China) and carried out in conformity with the ethical criteria outlined in the Helsinki Declaration in 1964. The patients/participants provided their written informed consent to participate in this study.

## Author Contributions

FC, BH, and JW participated in the design of the study. BH, LL, and YQ were responsible for recruiting participants. MG, YL, MY, LL, WH, and YuxW were responsible for interviewing participants. QD, YuyW, and HW analyzed the data. QD, YuyW, and XH wrote the manuscript, which was revised by all authors. All authors contributed to the article and approved the submitted version.

## Funding

This study was funded by the High-level Talents Research Start-up Project of Fujian Medical University (No. XRCZX2018001) and the Central Government-Led Local Science and Technology Development Special Project (No. 2020L3009).

## Conflict of Interest

The authors declare that the research was conducted in the absence of any commercial or financial relationships that could be construed as a potential conflict of interest.

## Publisher's Note

All claims expressed in this article are solely those of the authors and do not necessarily represent those of their affiliated organizations, or those of the publisher, the editors and the reviewers. Any product that may be evaluated in this article, or claim that may be made by its manufacturer, is not guaranteed or endorsed by the publisher.
