# Supplementary material for: Oolong Tea Consumption and the Risk of Oral Squamous Cell Carcinoma: A Propensity Score-Based Analysis in Southeast China
Source: Front Nutr. 2022 Jul 7;9:928840. doi: 10.3389/fnut.2022.928840 (PMC9301196; doi:10.3389/fnut.2022.928840)
Supplement: Supplementary file 1 [file Data_Sheet_1.docx]

Supplementary Material

Supplementary Table 1 Distribution and points assignment of oral hygiene indicators in the Overall population

| Variables | Overall (%) | Control (%) | Case (%) | *P* value | Points |  |
| --- | --- | --- | --- | --- | --- | --- |
|  |  |  |  |  |  |  |
| N | 1773 | 1029 | 744 |  |  |  |
| Teeth-brushing (times/day) |  |  |  | <0.001 |  |  |
| ＜1 | 81 (4.57) | 23 (2.24) | 58 (7.80) |  | 2 |  |
| 1 | 799 (45.06) | 416 (40.43) | 383 (51.48) |  | 1 |  |
| ≥2 | 893 (50.37) | 590 (57.33) | 303 (40.72) |  | 1 |  |
| Teeth lost |  |  |  | <0.001 |  |  |
| None | 637 (35.93) | 474 (46.06) | 163 (21.91) |  | 0 |  |
| <5 | 511 (28.82) | 304 (29.55) | 207 (27.82) |  | 1 |  |
| ≥5 | 625 (35.25) | 251 (24.39) | 374 (50.27) |  | 2 |  |
| Duration of denture (years) |  |  |  | <0.001 |  |  |
| No | 1054 (59.45) | 708 (68.80) | 346 (46.51) |  | 0 |  |
| ＜10 | 365 (20.59) | 173 (16.82) | 192 (25.80) |  | 1 |  |
| ≥10 | 354 (19.96) | 148 (14.38) | 206 (27.69) |  | 2 |  |
| Recurrent dental ulcer |  |  |  | <0.001 |  |  |
| No | 1650 (93.06) | 1012 (98.35) | 638 (85.75) |  | 0 |  |
| Yes | 123 (6.94) | 17 (1.65) | 106 (14.25) |  | 1 |  |
| Regular dental visits(times/year) |  |  |  | <0.001 |  |  |
| No | 1379 (77.78) | 728 (70.75) | 651 (87.50) |  | 1 |  |
| Yes | 394 (22.22) | 301 (29.25) | 93 (12.50) |  | 0 |  |
| Oral hygiene scores |  |  |  | <0.001 |  |  |
| 0 | 77(4.34) | 73(7.09) | 4(0.54) |  | \ |  |
| 1 | 318(17.94) | 253(24.59) | 65(8.74) |  |  |  |
| 2 | 393(22.17) | 276(26.82) | 117(15.73) |  |  |  |
| 3 | 306(17.26) | 169(16.42) | 137(18.41) |  |  |  |
| 4 | 297(16.75) | 150(14.58) | 147(19.76) |  |  |  |
| 5 | 231(13.03) | 79(7.68) | 152(20.43) |  |  |  |
| >6 | 151(8.51) | 29(2.82) | 122(16.39) |  |  |  |

Supplementary Table 2 Interaction analyses between oolong tea consumption and smoking or alcohol drinking for OSCC risk in overall population

| Variables | Oolong tea consumption | Control (%) n=1029 | Case (%) n=744 | Adjusted OR (95% CI) ^a^ | *P* for interaction |
| --- | --- | --- | --- | --- | --- |
| Smoking status ^b^ |  |  |  |  |  |
| No | No | 644(62.59) | 414(55.65) | 1.00 | 0.561 |
| No | Yes | 113(10.98) | 35(4.70) | 0.78(0.50-1.22) |  |
| Yes | No | 153(14.87) | 207(27.82) | 1.77(1.24-2.51) |  |
| Yes | Yes | 119(11.56) | 88(11.83) | 1.16(0.76-1.76) |  |
| Drinking status ^c^ |  |  |  |  |  |
| No | No | 697(67.74) | 457(61.42) | 1.00 | 0.672 |
| No | Yes | 144(13.99) | 50(6.72) | 0.75(0.50-1.11) |  |
| Yes | No | 100(9.72) | 164(22.04) | 2.27(1.59-3.23) |  |
| Yes | Yes | 88(8.55) | 73(9.81) | 1.49(0.97-2.29) |  |

a: Adjustment for age, gender, occupation, education level, BMI, residence, frequency of red meat, vegetables, and fruits; b: Additional adjustment for alcohol drinking; c: Additional adjustment for tobacco smoking. Abbreviation: OSCC, oral squamous cell carcinoma; OR (95% CI), the odds ratio and its 95% confidence interval.

Supplementary Table 3 Baseline characteristics of case and control groups before and after propensity score analyses

| Variables | | Overall (%) | Overall population | | |  | IPTW population | | |
| --- | --- | --- | --- | --- | --- | --- | --- | --- | --- |
|  |  |  | Control (%) | Case (%) | *P* value |  | Control (%) | Case (%) | SMD |
| N |  | 1773 | 1029 | 744 |  |  | 1788.6 | 1782.5 |  |
| Gender | Male | 955 (53.9) | 547 (53.2) | 408 (54.8) | 0.514 |  | 986.9 (55.2) | 991.4 (55.6) | 0.009 |
|  | Female | 818 (46.1) | 482 (46.8) | 336 (45.2) |  |  | 801.7 (44.8) | 791.0 (44.4) |  |
| Age (years) | < 60 | 1083 (61.1) | 729 (70.8) | 354 (47.6) | < 0.001 |  | 1072.8 (60.0) | 1087.4 (61.0) | 0.021 |
|  | ≥ 60 | 690 (38.9) | 300 (29.2) | 390 (52.4) |  |  | 715.8 (40.0) | 695.0 (39.0) |  |
| Occupation | Farmer | 439 (24.8) | 175 (17.0) | 264 (35.5) | < 0.001 |  | 458.5 (25.6) | 440.2 (24.7) | 0.034 |
|  | Worker | 271 (15.3) | 146 (14.2) | 125 (16.8) |  |  | 287.2 (16.1) | 273.2 (15.3) |  |
|  | Office worker and others | 1063 (60.0) | 708 (68.8) | 355 (47.7) |  |  | 1042.9 (58.3) | 1069.2 (60.0) |  |
| Education level | Illiteracy | 198 (11.2) | 106 (10.3) | 92 (12.4) | < 0.001 |  | 204.5 (11.4) | 213.0 (11.9) | 0.034 |
|  | Primary-middle school | 975 (55.0) | 486 (47.2) | 489 (65.7) |  |  | 994.9 (55.6) | 961.8 (54.0) |  |
|  | High school and above | 600 (33.8) | 437 (42.5) | 163 (21.9) |  |  | 589.2 (32.9) | 607.7 (34.1) |  |
| BMI | 18.5-23.9 | 1048 (59.1) | 576 (56.0) | 472 (63.4) | 0.002 |  | 1069.7 (59.8) | 1076.9 (60.4) | 0.013 |
|  | < 18.5 or ≥24 | 725 (40.9) | 453 (44.0) | 272 (36.6) |  |  | 718.9 (40.2) | 705.6 (39.6) |  |
| Residence | Rural | 772 (43.5) | 360 (35.0) | 412 (55.4) | < 0.001 |  | 801.0 (44.8) | 797.9 (44.8) | < 0.001 |
|  | Urban | 1001 (56.5) | 669 (65.0) | 332 (44.6) |  |  | 987.6 (55.2) | 984.5 (55.2) |  |
| Smoking status | No | 1206 (68.0) | 757 (73.6) | 449 (60.3) | < 0.001 |  | 1208.3 (67.6) | 1220.9 (68.5) | 0.02 |
|  | Yes | 567 (32.0) | 272 (26.4) | 295 (39.7) |  |  | 580.3 (32.4) | 561.5 (31.5) |  |
| Drinking status | No | 1348 (76.0) | 841 (81.7) | 507 (68.1) | < 0.001 |  | 1351.8 (75.6) | 1377.9 (77.3) | 0.041 |
|  | Yes | 425 (24.0) | 188 (18.3) | 237 (31.9) |  |  | 436.8 (24.4) | 404.6 (22.7) |  |
| Red meat intake | < 3 times | 970 (54.7) | 496 (48.2) | 474 (63.7) | < 0.001 |  | 994.2 (55.6) | 940.9 (52.8) | 0.056 |
| (per week) | ≥ 3 times | 803 (45.3) | 533 (51.8) | 270 (36.3) |  |  | 794.4 (44.4) | 841.6 (47.2) |  |
| Vegetable intake | ≥ 2 times | 622 (35.1) | 279 (27.1) | 343 (46.1) | < 0.001 |  | 616.9 (34.5) | 613.3 (34.4) | 0.002 |
| (per day) | < 2 times | 1151 (64.9) | 750 (72.9) | 401 (53.9) |  |  | 1171.7 (65.5) | 1169.2 (65.6) |  |
| Fruit intake | < 3 times | 899 (50.7) | 392 (38.1) | 507 (68.1) | < 0.001 |  | 923.9 (51.7) | 913.4 (51.2) | 0.008 |
| (per week) | ≥ 3 times | 874 (49.3) | 637 (61.9) | 237 (31.9) |  |  | 864.7 (48.3) | 869.0 (48.8) |  |

Abbreviation: PSM, propensity score matching; SIPTW, stabilized inverse probability of treatment weight; SMD, standardized mean differences.

Supplementary Table 4 The relationship between oolong tea drinking habits and OSCC risk in primary population and IPTW population

|  | Overall population | | |  | IPTW population | | |
| --- | --- | --- | --- | --- | --- | --- | --- |
| Variables | Control | Case | OR (95%CI) ^a^ |  | Control | Case | OR (95%CI) |
| Oolong tea consumption |  |  |  |  |  |  |  |
| No | 797(77.45) | 621(83.47) | 1.00 |  | 1408.5(78.75) | 1485.9(83.36) | 1.00 |
| yes | 232(22.55) | 123(16.53) | **0.67(0.50-0.90)** |  | 380(21.25) | 296.6(16.64) | **0.74(0.63-0.88)** |
| Average daily intake (ml/d) |  |  |  |  |  |  |  |
| Never drinking | 797(77.45) | 621(83.47) | 1.00 |  | 1408.5(78.75) | 1485.9(83.36) | 1.00 |
| < 500 | 90(8.75) | 54(7.26) | 0.75(0.49-1.14) |  | 148.6(8.31) | 123.2(6.91) | 0.79(0.61-1.01) |
| ≥ 500 | 142(13.80) | 69(9.27) | **0.62(0.43-0.89)** |  | 231.4(12.94) | 173.4(9.73) | **0.71(0.58-0.88)** |
| Duration of tea consumption (years) |  |  |  |  |  |  |  |
| Never drinking | 797(77.45) | 621(83.47) | 1.00 |  | 1408.5(78.75) | 1485.9(83.36) | 1.00 |
| < 20 | 124(12.05) | 32(4.30) | **0.45(0.28-0.71)** |  | 178.7(9.99) | 101.1(5.67) | **0.54(0.42-0.69)** |
| ≥ 20 | 108(10.50) | 91(12.23) | 0.85(0.60-1.22) |  | 201.4(11.26) | 195.5(10.97) | 0.92(0.75-1.13) |
| Age at onset of regular drinking (years) |  |  |  |  |  |  |  |
| Never drinking | 797(77.45) | 621(83.47) | 1.00 |  | 1408.5(78.75) | 1485.9(83.36) | 1.00 |
| < 30 | 93(9.04) | 72(9.68) | 1.06(0.72-1.56) |  | 150.9(8.43) | 187.6(10.52) | 1.18(0.94-1.48) |
| ≥ 30 | 139(13.51) | 51(6.85) | **0.43(0.29-0.64)** |  | 229.2(12.81) | 109(6.12) | **0.45(0.35-0.57)** |
| Tea temperature |  |  |  |  |  |  |  |
| Never drinking | 797(77.45) | 621(83.47) | 1.00 |  | 1408.5(78.75) | 1485.9(83.36) | 1.00 |
| Warm | 107(10.40) | 49(6.59) | **0.60(0.40-0.91)** |  | 176.2(9.85) | 130.5(7.32) | **0.70(0.50-0.99)** |
| Hot | 125(12.15) | 74(9.95) | 0.73(0.50-1.05) |  | 203.8(11.40) | 166(9.31) | 0.77(0.56-1.06) |
| Tea concentration |  |  |  |  |  |  |  |
| Never drinking | 797(77.45) | 621(83.47) | 1.00 |  | 1408.5(78.75) | 1485.9(83.36) | 1.00 |
| Light | 44(4.28) | 40(5.38) | 0.76(0.45-1.26) |  | 87.5(4.89) | 75.6(4.24) | 0.82(0.52-1.29) |
| Moderate | 130(12.63) | 51(6.85) | **0.54(0.37-0.81)** |  | 206(11.52) | 139.9(7.85) | **0.64(0.46-0.90)** |
| Strong | 58(5.64) | 32(4.30) | 0.91(0.54-1.55) |  | 86.6(4.84) | 81.1(4.55) | 0.89(0.57-1.39) |

a: Adjustment for age, gender, occupation, education level, BMI, residence, smoking and alcohol consumption, consumption frequency of red meat, vegetables, and fruits. Abbreviation: OSCC, oral squamous cell carcinoma; PSM, propensity score matching; SIPTW, stabilized inverse probability of treatment weight; OR (95% CI), the odds ratio and its 95% confidence interval.
